# Effective normalization for copy number variation detection from whole genome sequencing

**DOI:** 10.1186/1471-2164-13-S6-S16

**Published:** 2012-10-26

**Authors:** Angel Janevski, Vinay Varadan, Sitharthan Kamalakaran, Nilanjana Banerjee, Nevenka Dimitrova

**Affiliations:** 1 Philips Research, 345 Scarborough Rd, Briarcliff Manor, NY 10510, USA

## Abstract

**Background:**

Whole genome sequencing enables a high resolution view of the human genome and provides unique insights into genome structure at an unprecedented scale. There have been a number of tools to infer copy number variation in the genome. These tools, while validated, also include a number of parameters that are configurable to genome data being analyzed. These algorithms allow for normalization to account for individual and population-specific effects on individual genome CNV estimates but the impact of these changes on the estimated CNVs is not well characterized. We evaluate in detail the effect of normalization methodologies in two CNV algorithms FREEC and CNV-seq using whole genome sequencing data from 8 individuals spanning four populations.

**Methods:**

We apply FREEC and CNV-seq to a sequencing data set consisting of 8 genomes. We use multiple configurations corresponding to different read-count normalization methodologies in FREEC, and statistically characterize the concordance of the CNV calls between FREEC configurations and the analogous output from CNV-seq. The normalization methodologies evaluated in FREEC are: GC content, mappability and control genome. We further stratify the concordance analysis within genic, non-genic, and a collection of validated variant regions.

**Results:**

The GC content normalization methodology generates the highest number of altered copy number regions. Both mappability and control genome normalization reduce the total number and length of copy number regions. Mappability normalization yields Jaccard indices in the 0.07 - 0.3 range, whereas using a control genome normalization yields Jaccard index values around 0.4 with normalization based on GC content. The most critical impact of using mappability as a normalization factor is substantial reduction of deletion CNV calls. The output of another method based on control genome normalization, CNV-seq, resulted in comparable CNV call profiles, and substantial agreement in variable gene and CNV region calls.

**Conclusions:**

Choice of read-count normalization methodology has a substantial effect on CNV calls and the use of genomic mappability or an appropriately chosen control genome can optimize the output of CNV analysis.

## Background

Genetic variation in the human genome occurs in many forms ranging from large chromosomal abnormalities to single nucleotide variations, each with varying functional significance. Copy number variation (CNV) is one such genetic variation that can range from a few kilobases to megabases, and involve deletions, duplications, insertions or translocations. Multiple methodologies on a genome-wide scale have been used, including RDA, CGH, and more recently: CNV calling using next generation sequencing [1]. The associations between CNVs and phenotypic variation or disease-susceptibility are increasingly being investigated [2], with the most obvious mechanism being gene-dosage caused by variations in the number of copies of a gene or its associated regulatory elements. However, investigations into the biological implications of CNVs in normal and cancer samples have largely been limited by measurement technologies. Multiple studies have confirmed that the amplifications of oncogenes and loss of tumor suppressors are implicated in the development and progression of cancer. Validation of CNV results has been extremely challenging due to natural CNV variations within and across populations [3-5]. This problem is even more exacerbated in cancer, and it is now recognized that cancers have CNV subtype profiles [6]. Establishing frameworks for evaluation of CNV algorithms is very important both for human diversity studies as well as cancer.

In contrast to microarray-based CNV detection methods, where probes are designed following a carefully developed protocol, whole genome sequencing reads represent a random sampling from a library and could be susceptible to biases, for example GC content and other biophysical and chemical characteristics. In addition, the structure of the human genome with repeat elements and paralogous stretches of sequences make mapping of individual sequencing reads to a reference genome a non-trivial problem.

Several methods and tools have addressed various aspects of determining CNV information based on sequencing data, each accounting for different forms of normalization: for example, CNVnator [7] uses a mean-shift approach with additional refinements (multiple-bandwidth partitioning and GC correction), CNV-seq produces relative copy number profiles from paired genomes with one serving as a reference [8], FREEC uses mappability and a control genome [9], and an interesting application of mrFAST and msFAST [3]. Each tool has a unique approach and is successful in addressing some of the CNV detection challenges. Of the available CNV estimation tools, we selected FREEC and CNV-seq as both tools apply a statistical approach to integrate background information about the genome into making CNV calls. Additionally, FREEC allows for various types of normalizations, which we deemed a key element in analyzing genome sequences from multiple populations. The FREEC tool was developed specifically to enable control-free copy number alteration detection [9]. FREEC requires the user to only provide the ploidy of the genome in order to assign absolute copy number to each predicted CNV, but FREEC can also be run in other modes which allow for the normalization of read counts in the sample genome by inclusion of a control genome or a mappability track. The algorithm first calculates a raw copy number profile by counting the number of reads that map to non-overlapping windows across the entire genome. The second step involves normalizing these raw read counts to account for sequence characteristics of the genome that could influence the number of reads within each window, followed by segmentation and subsequent copy-number estimations.

We apply FREEC on a set of genomes spanning four populations to assess concordance between the outputs from different normalization methods. We also analyze the same genomes with CNV-seq and we compare the output from both tools.

## Methods

### Whole genome sequencing data

To characterize the landscape of structure variations, we use whole genome sequencing data from three publicly available genomes of Caucasian (CEU, NA12891; CEf, NA12892 [4]), African (YRI1, NA19239 [6]; YRI2 NA 18507 [10]), and Asian (Yh1 [11]; KOR [12]) individuals, as well as two currently unpublished genomes of two individuals (UG1, and UG2) from a population distinct from the six public genomes. All were sequenced using the Illumina GAII sequencer using paired-end and single-end reads differing primarily in the read length - the first three sets genomes are based mostly on 36-base reads, whereas the 2 additional genomes are using 115 base reads. All genomes were processed and aligned for this analysis using the Burrows Wheelers Aligner [13] and Samtools [14] to 25x coverage or higher using a total number of aligned bases ranging from 100 to 275 million bases. Identical assembly pipeline was applied to align the genomes to the Hg19 human genome reference. For validation, we use genic regions as defined in RefGene [15], as well as a 8,599 validated CNV segments from Conrad et al. [5] comparing genotyping information from several populations.

### Estimating copy number variation with FREEC

We run FREEC with 3 Kb window size and other default parameters except those under study (detailed below). We compared the CNV estimates from the output of FREEC, which applies a sliding window strategy with GC content normalization to make absolute copy number predictions. FREEC first maps reads from a given sequencing run to non-overlapping windows spanning the entire reference genome. The raw copy number of a given genomic region is assumed to be proportional to the number of reads that align to the windows spanning that region. The algorithm then normalizes these raw read counts to account for sequence characteristics of the genomic region which influence the number of reads within each window. A segmentation algorithm is applied to the normalized read counts to identify contiguous windows that make up a genomic region with a unique copy-number value. The final step in the algorithm estimates the copy-number value of the segmented genomic regions, thus resulting in a genome-wide copy number profile.

FREEC can be run in three configurations corresponding to different normalizations of read counts within a window [see Additional File 1]. We compare FREEC results from the different configurations with CNV estimation based on normalization from: (i) GC content, (ii) mappability [16] (76-base segment length for UG1 and UG2, and 36-base segment length for the remaining genomes), and (iii) control genome.

Mappability characterizes the degree to which a region of certain length is distinct and hence uniquely mappable to the reference genome. We use FREEC's default parameter which, given mappability information, considers regions for which 85% of the bases are mappable. We use three different criteria to select a control genome: (i) in-population where all genomes use as control the other genome from the same population, (ii) single control genome YRI1 (African, similar coverage profile as most genome), and (iii) single control genome YRI2 (African, high coverage genome, >50X). For in-population controls, the genomes are paired as follows: UG1 and UG2, YRI1 and YRI2, CEU and CEf; and Yh1 and KOR.

### Estimating copy number variation with CNV-seq

CNV-seq [8] uses a fixed length sliding window and normalization of the analyzed (test) genome using a control genome. Differences in read counts mapped between the control and test genomes for a given segment determine the relative copy number change. We use CNV-seq with two modifications of the default parameters for increased stringency: lower p-value for the CNV calls (10^-4 ^instead of the default 10^-3^), and higher number of consecutive windows required to call a variation (6 instead of the default 4). We present results using a 3 kilobase window and the trends remain the same with smaller or larger window sizes (data not shown).

We use two sets of seven CNV-seq comparisons, where CNV analysis was done using YRI1 in the first and YRI2 in the second set. These sets are analogous to the FREEC configurations using the African genomes as control, namely (ii) and (iii) in the previous subsection.

### Merging CNV calls into comparative CNV calls

**Figure 1:**
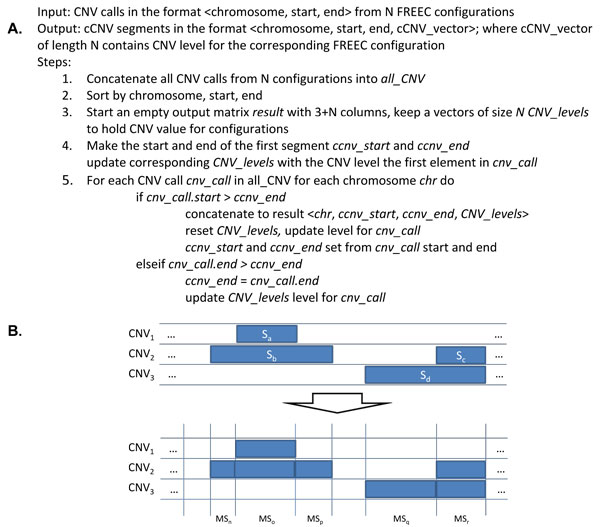
**cCNV: Merging of CNV segments from multiple FREEC configurations**. A. cCNV algorithm. B. Example of merging CNV calls.

To enable comparative analysis of CNV segments output by multiple tool configurations, we consolidate the outputs of each of the three FREEC configurations and one CNV-seq configuration into a set of CNV segments that characterize variations collectively. The steps in the method for merging the CNV segment are given in Figure 1A, and are also illustrated with an example in Figure 1B. Briefly, a merged list of CNV segments is generated by aligning all detected CNV segments in a set of genomes and extracting all unique segment start and end positions to generate a new, more granular set of CNV regions. CNV values are assigned each to these regions for each genome, based on the overlap of the new segments with the original CNV calls. The merging of segments generates an aggregated view of a set of CNVs, and effectively adds a "second dimension" to the genome resulting from a combined set of genomes, represented with a vector containing the original CNV values.

In the example in Figure 1B, the CNV calls from three configurations are broken into five merged segments MS_n _- MS_r _each of which is characterized with a vector of three values. We call these segments comparative CNV (cCNV) regions. In the example in Figure 1, two cCNV are formed (MS_n_-_r _and MS_p_-_q_) as a result of this step.

The creation of cCNV segments tends to "pad" the segments in one or more CNV estimates as the segments are merged to meet the outer boundaries of overlapping CNV segments. In our current approach, we focus on encompassing all bases in a shared CNV region. In a more conservative approach, one may consider a more restrictive method to prune the ends of cCNV segments and narrow the overlapping segment. In our approach, due to the tendency of FREEC to merge very large adjacent segments into very long segments (several million bases), we introduce a pre-processing step to fragment segments into 10,000-base segments and avoid superficial extension of cCNV segments into configurations where a small region was originally detected. In our example, this would remedy a case where the length of S_c _is relatively small, and S_d _very large. With the fragmentation step, S_d _is split into k segments: S_d1_, S_d2_,... S_dk _and only a subset of these overlapping with Sc are returned as an overlapping cCNV region for CNV_2 _and CNV_3_. The remaining S_di _are separately considered as a variation only in CNV_3_.

In a subsequent step, all cCNV segments are annotated for their overlap with the gene regions in refSeq and the CNV regions in the [5], referred to as 'Sanger' in the rest of the text. Based on the overlap, each cCNV region is assigned two values between 0 and 1 ranging between no overlap (0), partial overlap (between 0 and 1), and full overlap (1).

To obtain the number of bases in genic regions, we use the following formula: $Genic\left(c\right)=\underset{c\;\in \;cCNV\;regions}{\sum }length\left(c\right)*refSeq\text{\_}overlap\left(c\right)$where for each cCNV region *c*, we scale its length given in number of bases with the fraction of the segment overlapping with the gene (*refSeq_overlap*). Non-genic regions are simply the inverse of genic regions: $NonGenic\left(c\right)=\underset{c\;\in \;cCNV\;regions}{\sum }\left[length\left(c\right)-Genic\left(c\right)\right]$. Finally, to obtain the number of bases overlapping Sanger regions, we use: $Sanger\left(c\right)=\underset{c\;\in \;cCNV\;regions}{\sum }length\left(c\right)*Sanger\text{\_}overlap\left(c\right)$, where *Sanger_overlap *indicates the fraction of the segment overlapping with Sanger regions.

### Comparison of configurations and estimating concordance

**Figure 2:**
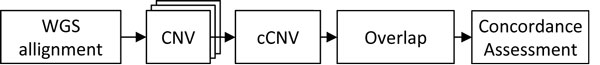
**Comparison of analysis outputs**. Our analysis pipeline which compares the results from analyzing results from three FREEC modes. cCNV merges all the output regions for the three. cCNV segments are characterized with respect to coverage and overlap overall, in genic and non-genic regions, and in the context of the 8,599 validated segments from [11].

Figure 2 depicts our analysis flow which compares the results from the execution of FREEC and CNV-seq. After Alignment, the CNV step applies a selected FREEC or CNV-seq configuration resulting in CNV calls. The cCNV step merges all the output segments from the configurations. cCNV segments are characterized in the Overlap step to quantify the intersection with genic regions, and 8,599 validated segments from the Sanger set. In the last step, Concordance Assessment, we assess concordance between the three FREEC normalizations. We measure the similarity using the Jaccard index (JI) on any two sets of output comparing the sets at the level of individual base pairs. Given two configurations A and B, we use the following to compute Jaccard Index: $JI\left(conf_{A},conf_{B}\right)=\frac{\left|conf_{A}\cap conf_{B}\right|}{\left|conf_{A}\cup conf_{B}\right|}$. It computes the ratio of the total number of cCNV bases in the intersection of configurations A and B over total number of bases in the union of the of the two sets JI is reported for each genome and four CNV segment sets: all regions, genic, non-genic, and Sanger.

### Variation profiles of cCNV

Additionally, for the output of FREEC, the number of bases falling under a range of copy number amplifications and deletions is reported for individual genomes and within segment sets. The output of this characterization is binned using the following breakpoints: less than 2 copies, 2 to 6 copies, and more than 6 copies.

## Results

### cCNV regions

We focus only on the autosomal CNV segments (chromosomes 1 through 22). We transform the CNV regions called by FREEC to cCNVs. We interchangeably refer to CNV and cCNV regions in the remainder of the text - all discussion refers to the CNV obtained as a result of combining CNV regions from FREEC configurations, i.e. one should consider all CNV references to be cCNV. Table 1 provides a summary of the output from three separate control genome choices for FREEC and Table 2 provides a summary for the output of CNV-seq. In FREEC, GC content normalization outputs CNV regions ranging 57-98 Mb among the eight genomes. Adding mappability as a normalization parameter results in a substantially reduced total number of bases called in comparison to GC content normalization - calling only 25% of the total base pairs outputted by GC normalization but going as low as 6-7% in three genomes (CEU, YH1, and KOR). Adding an in-population control genome as normalization parameter in FREEC yields a smaller reduction of the total lengths ranging from 25 to 60% with one exception (YRI2, with an over 2-fold increase in total lengths). With YRI1 as a control genome, the total length of base pairs in the control configuration is in the 60% range for most genomes, with Yh1 at 82%, and CEf at 95%, of the GC content normalization total length, except for an over 2-fold increase with YRI2. Using YRI2 as a control results in a number of variant base pairs comparable to the GC content normalization total length for most genomes, and around 70% of the total length in YRI1 and CEf. Using YRI1 or YRI2 as a control genome yields to similar summaries in case of mappability and GC content normalization. We observe ~10% variation due to the "padding" effect discussed earlier.

**Table 1:** 

FREEC Normalization	UG1	UG2	YRI1	YRI2	CEU	CEf	Yh1	KOR
GC content	77,347,858	79,636,676	97,902,881	70,686,285	76,746,044	89,623,788	78,456,632	57,134,301
Mappability	19,520,720	17,252,109	24,758,352	17,768,115	4,925,758	20,064,719	5,697,806	3,931,768
Control genome (in Population)	37,100,227	20,683,564	67,291,545	163,831,511	48,113,551	32,010,850	50,189,736	33,322,468
Control genome (YRI1)	41,084,695	37,640,839	-	163,831,511	52,945,367	87,318,862	50,727,044	38,000,863
Control genome (YRI2)	117,640,623	74,253,250	67,291,545	-	58,461,174	62,120,940	69,839,893	78,225,240

**Table 2:** 

CNV-seq Normalization	UG1	UG2	YRI1	YRI2	CEU	CEf	Yh1	KOR
Control genome (YRI1)	43,235,491	42,881,033	-	63,065,487	22,266,758	27,151,018	25,818,765	38,632,528
Control genome (YRI2)	113,662,351	106,834,661	108,082,096	-	59,313,018	49,010,115	79,994,365	43,093,200

In CNV-seq, the total length of CNV regions ranging 22-63 Mb with YRI1 as a control genome and 43-113 Mb with YRI2 as a control genome. These values are comparable to the ranges in the analogous FREEC runs with control genome normalization.

We then classify the called segments as genic, non-genic and also compare them to a list of commonly occurring variant regions previously reported [3]. Table 3 shows the representation of genic segments in the cCNV regions. In FREEC as shown in Table 4, genic regions on average constitute 49.7% (sd = 17.5%) in mappability, 17.5% (sd = 5.8%) in GC content, and 27.9% (sd = 15.7%) in control genome normalization. As we change the control genome to YRI1 or YRI2, similar distribution for genic regions is observed for mappability normalization. However, in control normalization, the impact varies: more genic regions are returned in UG1, CEU, and Yh1, but mappability still has more enrichment in genic regions for all genomes.

**Table 3:** 

CNV-seq Normalization	UG1	UG2	YRI1	YRI2	CEU	CEf	Yh1	KOR
Control genome (YRI1)	46%	46%	-	55%	57%	53%	61%	56%
Control genome (YRI2)	55%	55%	59%	-	56%	56%	54%	48%

**Table 4:** 

FREEC Normalization	UG1	UG2	YRI1	YRI2	CEU	CEf	Yh1	KOR
GC content	13%	12%	28%	20%	12%	24%	11%	21%
Mappability	30%	29%	79%	51%	48%	80%	36%	45%
Control genome (in Population)	10%	21%	37%	51%	14%	56%	13%	21%
Control genome (YRI1)	22%	21%	-	51%	21%	38%	17%	25%
Control genome (YRI2)	26%	24%	37%	-	17%	32%	22%	23%

Table 5 shows the representation of Sanger regions in the total cCNV regions. In FREEC as shown in Table 6, Sanger regions on average account for 14.9% (sd = 4.9%) in mappability, 23% (sd = 5.7%) in GC content, and 22.4% (sd = 6.8%) in control genome normalization. Using YRI1 or YRI2 as a control genome, again similar trends are observed for mappability normalization. In control normalization, YRI1 as control increases the representation of these regions in UG1. Using YRI2 as a control substantially reduces the representation of these regions for KOR.

**Table 5:** 

CNV-seq Normalization	UG1	UG2	YRI1	YRI2	CEU	CEf	Yh1	KOR
Control genome (YRI1)	34%	34%	-	29%	41%	43%	37%	27%
Control genome (YRI2)	24%	24%	24%	-	28%	34%	26%	31%

**Table 6:** 

FREEC Normalization	UG1	UG2	YRI1	YRI2	CEU	CEf	Yh1	KOR
GC content	19%	21%	18%	25%	21%	19%	23%	38%
Mappability	19%	19%	5%	16%	12%	9%	18%	21%
Control genome (in Population)	15%	28%	20%	12%	22%	21%	23%	37%
Control genome (YRI1)	31%	32%	-	12%	20%	17%	20%	36%
Control genome (YRI2)	15%	22%	20%	-	25%	19%	24%	26%

### Effect of using mappability track on copy number estimation

**Figure 3:**
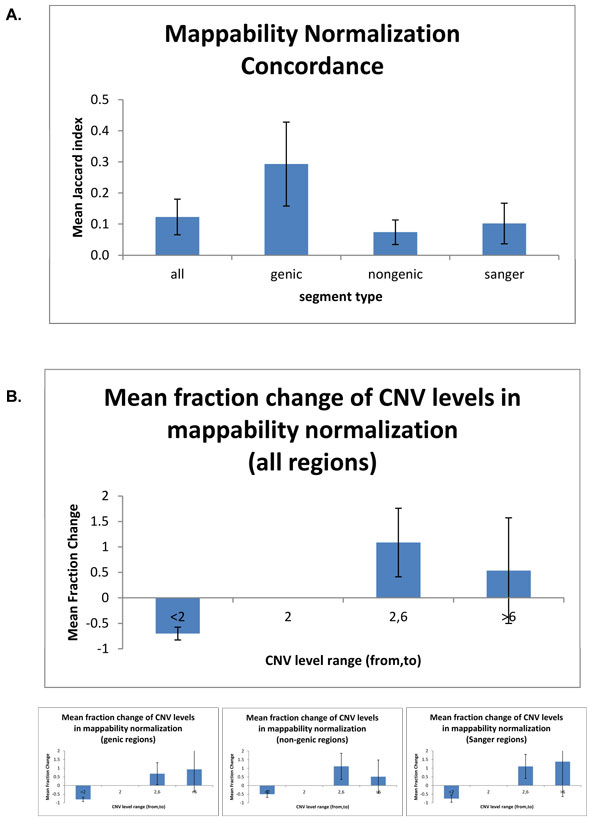
**Summary of mappability concordance**. A. Summary of mappability concordance for the eight genomes given separately for all, genic, non-genic, and Sanger regions. cCNVs are computed using in-population control. B. Mean fraction change with mappability normalization at three levels: below 2 copies, 2-6 copies and more than 6 copies.

Using mappability tracks significantly alters the number of copy number variants reported. We calculate the Jaccard Index as a measure of similarity between the reported CNVs by FREEC using mappability normalization over GC content normalization. Averaged over the eight genomes, the JI was 0.123 (sd = 0.057), indicating low levels of commonality between the reported CNVs (Figure 3A). We annotate the reported calls as genic, non-genic and investigate any differences. Interestingly, JI is higher in genic regions (mean = 0.29, sd = 0.135) than in non-genic regions (mean = 0.074, sd = 0.039), with a significant difference (p-value = 0.0006, Wilcoxon rank sum test). Next, we investigate whether the amplitude of copy number calls varies when mappability is used in normalization (Figure 3B, individual cCNV data is shown in Supplementary Figure 3 [see Additional File 2]). We find significant differences between the reported calls when mappability is used. Using mappability causes a fraction change in the number of CNV deletions reported by FREEC and shift towards higher copy number calls. On average, 70% of the deletions disappear consistently across the 8 individual genomes (sd = 12.5%). The higher copy number in the range of 2 to 6 copies increases by 108% in a highly variable fashion (sd = 67%). Most of these regions appear to be from non-genic areas of the genome.

### Effect of using a control genome on copy number estimation

**Figure 4:**
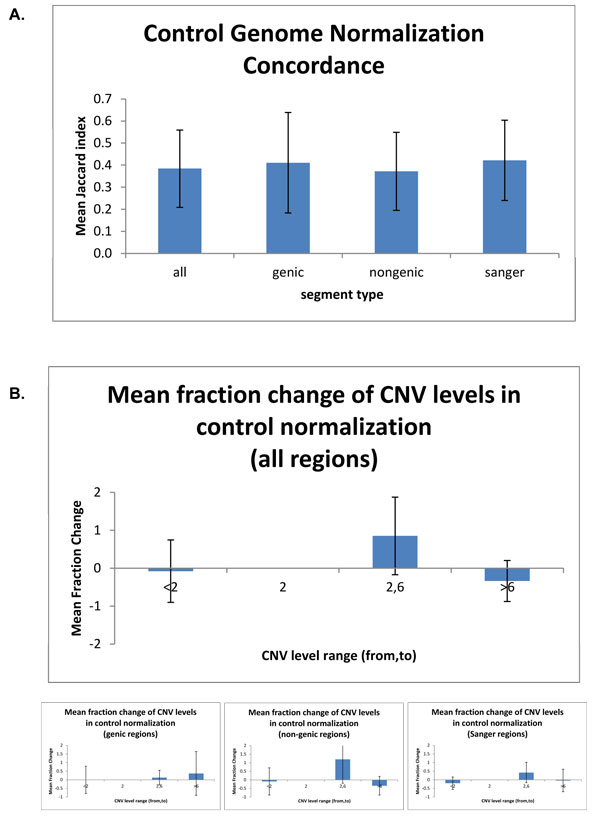
**Summary of control genome concordance**. A. Summary of control concordance for the eight genomes given separately for all, genic, non-genic, and Sanger regions. cCNVs are computed using in-population control.B. Mean fraction change with control normalization at three levels: below 2 copies, 2-6 copies and more than 6 copies.

We observe higher similarity between the copy number variants reported when a control genome is used for normalization as opposed to the mappability criterion shown above. The JI between normalization using a control genome and GC content normalization is 0.384 (sd = 0.176) when averaged over 8 genomes, with similar values within genic and non-genic annotations of the genome (Figure 4A, also Supplementary Figure 4B and 4C [see Additional File 2]). There is no consistent change in the numbers of called deletions or high amplifications between using a control genome and GC content. We observe 0-mean for the fractional change for all regions, with high variability (sd = 82.2%). The fraction changes given in Figure 4B (see also, Supplementary Figure 3 [see Additional File 2]) show the copy number range from 2 to 6 exhibiting high fractional change (mean = 85.3%) and great variability (sd = 102.1%). This change is most notable in the non-genic regions (mean = 120.2% and sd = 139.4%). Even more striking is variability in the fractional change of the high copy number in the genic regions (mean = 36.6%, sd = 127.8%).

In CNV-seq, genic regions are represented in levels comparable to the levels of FREEC with mappability: 53.3% (SD = 5.2%) for YRI1 as a control, and 54.7% (sd = 3.25%) for YRI2 as a control. Furthermore, we observe higher representation of Sanger regions: 35% (sd = 5.4%) with YRI1 as control, and 27.2% (sd = 3.6%) with YRI2 as control).

## Discussion

The findings presented here have established that a calculation of CNV in whole genome sequencing data without proper normalization with a control genome can be misleading. Much attention needs to be paid to the type of sample, context of the analysis and the population represented in the genome as they may have profound effects on the number of CNV calls. Major challenges in the CNV calling come from the technical difficulty to map regions of the genome with lower mappability and account for repeat regions in a balanced manner. We show that FREEC applied with mappability normalization results in lower concordance between the reported CNVs when compared to GC based normalization. There is enrichment of CNVs in genic regions - in most individuals genic CNVs encompass about 30-50% of the total CNVs (and they represent about 1% of the genome). Furthermore, in mappability normalization, there is significantly higher concordance in the genic regions which could be due to the fact that the mappability of genic regions is typically higher. The coarse definition of mappability in FREEC considers equally all bases with non-zero mappability score is better suited for regions that exhibit higher mappability. Therefore, with non-genic regions likely to have lower mappability scores, this criterion filters out fewer non-genic regions that are overall with lower mappability.

We observe that GC content based normalization in FREEC is likely not accounting for the expected lower number of reads within regions of lower mappability. Algorithms that attempt to call absolute copy number are susceptible to deletion-bias since they typically compare read-counts in specific regions against the background read-count distribution across the whole genome within a single sequencing run. Algorithms implicitly assume that reads are generated uniformly from across the whole genome with read-counts within any given window therefore following a Poisson distribution [4]. However, it is essential to note that while reads are likely *generated *uniformly from across the genome, they are not necessarily *mapped *uniformly across the genome even in a purely diploid sample. Regions of the genome with lower mappability tend to accumulate lower read-counts, thus leading to read-count artifacts that can be interpreted as deletions in the absence of proper read-count normalization techniques. Algorithms that attempt to call relative copy numbers, such as CNV-seq, are less prone to mappability artifacts although they suffer from run-specific sources of read-count variability. Indeed, 99% of the output from CNV-seq is regions that FREEC considers highly-mappable.

**Figure 5:**
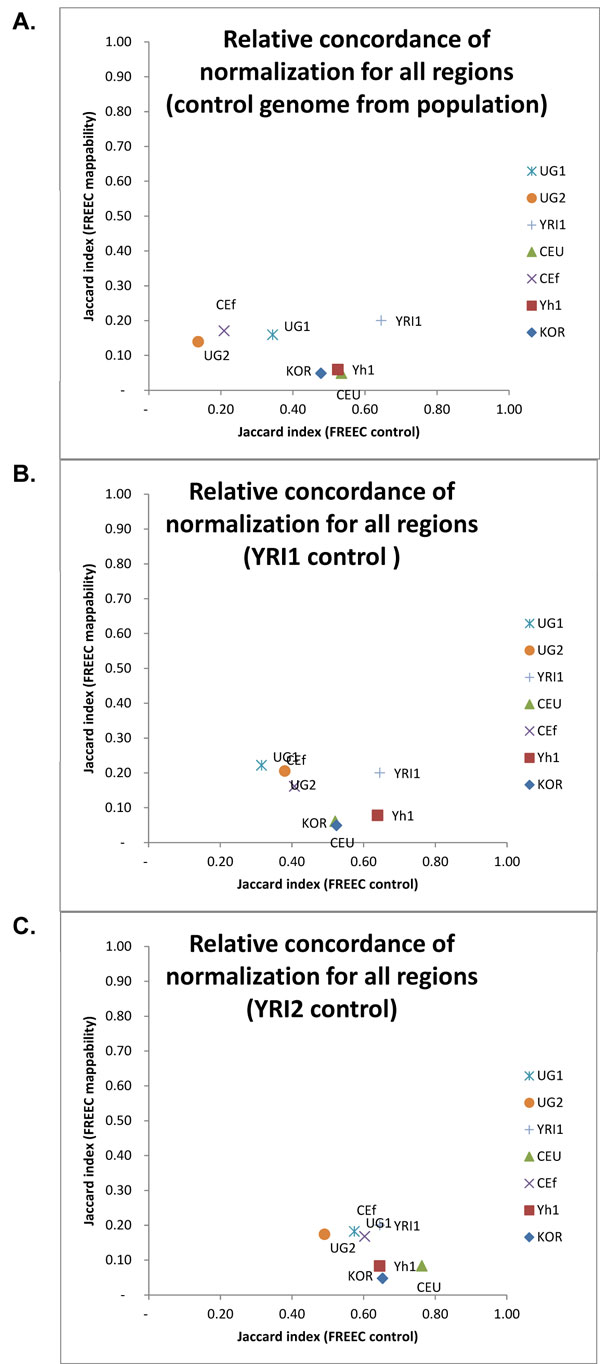
**Relative concordance for all CNV regions**. A. Jaccard indices of control vs. mappability normalization - control genome from population. B. Jaccard indices of control vs. mappability normalization - control genome isYRI1C. Jaccard indices of control vs. mappability normalization -control genome is YRI2.

The use of a control genome to normalize the read-counts could be thought of as a possible surrogate for a mappability track since presumably regions of poor mappability wouldn't change from the sample genome to the control genome. Furthermore, it could be hypothesized that the control genome ought to be chosen from within the same population as the sample genome. For the FREEC output, we estimated the Jaccard similarity indices of the control-genome based CNV calls with the GC content based CNV calls for each of the 8 samples by using an in-population control genome and compared these with the respective values of the mappability based CNV calls (Figure 5A and Supplementary Figure 5 [see Additional File 2]). We observe increased variation in the JI values for in-population control genome based normalization when compared to the mappability based normalization. Replacing the in-population control genome with a single control genome for all samples, such as the use of YRI1 as a universal control, results in relatively smaller variation in JI values (Figure 5B). Furthermore, choosing a universal control with much higher average coverage of the genome than the sample yields higher similarity of CNV profiles to the GC content normalization (Figure 5C). In other words, if the aim is to maintain higher agreement with the CNV calls out of the GC content normalization procedure, a control genome with similar average coverage as the sample genome (YRI1) might be preferable to a control genome with much higher average coverage (YRI2). Further investigation is needed to determine whether the choice of control genomes affects false positive rates. Relative concordance is worst when we look at the genic regions (Supplementary Figure 5 [see Additional File 2]) and in-population control, both mappability and population-specific variations contributing to this effect. Mappability restricts the concordance most tightly in the Sanger regions - possibly due to the fact that these regions have been already mapped and validated in many individuals - so the individual and population variability affects these results the least.

Finally, when the output of FREEC is compared to the output of CNV-seq, we observe that an explicit normalization (a control genome), also introduces an implicit mappability normalization to the extent of FREEC's use of mappability. The level of agreement in terms of called variant genes and Sanger CNVs between FREEC and CNV-seq are given in Supplementary Tables 1 and 2 [see Additional File 2]. While we observe comparable CNV call profiles between effectively normalized FREEC and CNV-seq, typically CNV-seq would call in the order of 40-50% of the genes or regions called by FREEC.

## Conclusions

We observe the effects of adding mappability and control genomes for normalization and we find that both types of normalization improve on the CNVs called by FREEC, with similar CNV call profiles observed in CNV-seq. Extending other tools with similar capability to normalize the analyzed sequence would likely yield similar improvement in concordance. The improvement in concordance is evident with control genome normalization in FREEC and to some extent with mappability normalization. We have some reservation with FREEC's current mappability normalization, as it does not filter out all regions with lower mappability scores - a more elaborate criterion for the mappability filter (e.g. average mappability score) we believe should yield performance comparable to control normalization. Control genome normalization typically introduces more new information to the CNV calls especially in the higher copy number regions. This effect is smaller in genic regions and in majority of the genomes when considering the validated Sanger segments.

The implication of our analysis is that CNV estimation algorithms that use sequencing data should extend their methods to account for mappability and other sources of variation in the context of multiple sequences and integrate this input into CNV call assessments. Notably, steps should be taken to normalize the output in the context of the population of origin as studies show population-specific variations that may not be accounted for by generic normalization approaches.

Understanding and optimizing CNV methods and tools will be essential as whole genome sequencing will be entering clinical practice in the near future with the promise of characterizing tumor samples down to a single cell. Robust and repeatable methods will be essential for accurate tracking of the progression of cancer and also characterization in the context of other sampled tumors.

## Competing interests

The authors declare that they have no competing interests.

## Authors' contributions

AJ implemented and executed FREEC and cCNV generation and annotation; ND/AJ executed the whole genome assembly and coverage computation. All authors contributed in the analysis and interpretation of the result and writing the text.

## Supplementary Material

Additional file 1 **FREEC normalization options**. Normalization in FREEC. Click here for file

Additional file 2 **Supplementary figures and tables**. Additional figures and tables referenced in the main document. Click here for file
