# Supplementary material for: Effective normalization for copy number variation detection from whole genome sequencing
Source: BMC Genomics. 2012 Oct 26;13(Suppl 6):S16. doi: 10.1186/1471-2164-13-S6-S16 (PMC3481445; doi:10.1186/1471-2164-13-S6-S16)
Supplement: Additional file 1 — FREEC normalization options. Normalization in FREEC. [file 1471-2164-13-S6-S16-S1.doc]

## FREEC Normalization Options

The FREEC tool was developed specifically to enable control-free copy number alteration detection [1]. FREEC requires the user to only provide the ploidy of the genome in order to assign absolute copy number to each predicted CNA, but FREEC can also be run in other modes which allow for the normalization of read counts in the sample genome by inclusion of a control genome or a mappability track. The algorithm first calculates a raw copy number profile by counting the number of reads that map to non-overlapping windows across the entire genome. The second step involves normalizing these raw read counts to account for sequence characteristics of the genome that could influence the number of reads within each window, followed by segmentation and subsequent copy-number estimations. FREEC allows for the following three types of normalization of the read counts within a given window:

***Normalization using GC content (FREEC without mappability or control)*:** Given the sample ploidy *P*, which in our case equals 2 for all the genomes analyzed, FREEC models the observed read count in *2*-copy regions as a 3^rd^ degree polynomial of the GC content of the associated windows. The polynomial
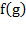
 is fit at GC content values in increments of 15% from 35% to 55%. In order to fit the polynomial, FREEC uses an iterative process where it first attempts to find windows that fall into 2-copy regions by assigning a copy number for the *i^th^* window as
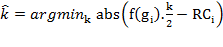
, and then fits the polynomial
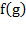
using all windows assigned a copy number of 2. This process is repeated until the parameters of the polynomial converge. Upon convergence, the normalized read-count in window *i* is calculated as:
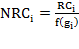
.

***Normalization using a control genome*:** When FREEC is run with a control genome, the read count within each window is normalized by a cubic polynomial function of the read count in the same window from the control genome. The parameters of this polynomial
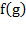
 are estimated using all windows assuming zero intercept. The normalized read counts are calculated as
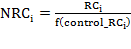
.

***Normalization using a mappability track*:** When FREEC is run with a mappability track, FREEC augments the polynomial estimated using the window’s GC content with the fraction of uniquely mappable positions per window as:
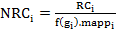
.

**References:**

1. Boeva V, Popova T, Bleakley K, Chiche P, Cappo J, Schleiermacher G, Janoueix-Lerosey I, Delattre O, Barillot E: **Control-free calling of copy number alterations in deep-sequencing data using GC-content normalization**. *Bioinformatics 2011*, **27(2)**:268-9
