# Supplementary material for: Effective normalization for copy number variation detection from whole genome sequencing
Source: BMC Genomics. 2012 Oct 26;13(Suppl 6):S16. doi: 10.1186/1471-2164-13-S6-S16 (PMC3481445; doi:10.1186/1471-2164-13-S6-S16)
Supplement: Additional file 2 — Supplementary figures and tables. Additional figures and tables referenced in the main document. [file 1471-2164-13-S6-S16-S2.doc]

## Supplementary Figure 1A

## Supplementary Figure 1B

## Supplementary Figure 2A

## Supplementary Figure 2B

## Supplementary Figure 3

| All regions | Genic regions |
| --- | --- |
|  |  |

## Supplementary Figure 4A

## Supplementary Figure 4B

## Supplementary Figure 4C

## Supplementary Figure 5A

##

## Supplementary Figure 5B

## Supplementary Figure 5C

## Supplementary Table 1

|  |  | GC Content | | Mappability | | Control Genome | | Variable genes in FREEC called by CNV-seq [%] | | |
| --- | --- | --- | --- | --- | --- | --- | --- | --- | --- | --- |
| Genome | CNV-seq | FREEC | FREEC & CNV-seq | FREEC | FREEC & CNV-seq | FREEC | FREEC & CNV-seq | GC Content | Mappability | Control Genome |
| UG1 | 1,221 | 212 | 122 | 657 | 369 | 377 | 224 | 61% | 60% | 57% |
| UG2 | 1,159 | 206 | 103 | 714 | 385 | 370 | 222 | 55% | 57% | 53% |
| YRI1 | 1,073 | 218 | 173 | 813 | 428 | 1,208 | 621 | 83% | 71% | 58% |
| CEU | 678 | 45 | 14 | 693 | 224 | 493 | 186 | 48% | 36% | 49% |
| CEf | 646 | 127 | 71 | 746 | 300 | 327 | 184 | 68% | 59% | 63% |
| Yh1 | 608 | 73 | 26 | 738 | 249 | 557 | 210 | 29% | 33% | 34% |
| KOR | 935 | 42 | 20 | 819 | 363 | 543 | 249 | 35% | 35% | 36% |

## Supplementary Table 2

|  |  | GC Content | | Mappability | | Control Genome | | Variable genes in FREEC called by CNV-seq [%] | | |
| --- | --- | --- | --- | --- | --- | --- | --- | --- | --- | --- |
| Genome | CNV-seq | FREEC | FREEC & CNV-seq | FREEC | FREEC & CNV-seq | FREEC | FREEC & CNV-seq | GC Content | Mappability | Control Genome |
| UG1 | 1,221 | 212 | 122 | 657 | 369 | 377 | 224 | 58% | 56% | 59% |
| UG2 | 1,159 | 206 | 103 | 714 | 385 | 370 | 222 | 50% | 54% | 60% |
| YRI1 | 1,073 | 218 | 173 | 813 | 428 | 1,208 | 621 | 79% | 53% | 51% |
| CEU | 678 | 45 | 14 | 693 | 224 | 493 | 186 | 31% | 32% | 38% |
| CEf | 646 | 127 | 71 | 746 | 300 | 327 | 184 | 56% | 40% | 56% |
| Yh1 | 608 | 73 | 26 | 738 | 249 | 557 | 210 | 36% | 34% | 38% |
| KOR | 935 | 42 | 20 | 819 | 363 | 543 | 249 | 48% | 44% | 46% |
